# From Surge to Stability: Telemedicine Use in Urology Four Years After the Pandemic

**DOI:** 10.7759/cureus.90885

**Published:** 2025-08-24

**Authors:** Mandy Hsu, Jay D Raman

**Affiliations:** 1 Urology, Penn State College of Medicine, Hershey, USA; 2 Urology, Penn State Health Milton S. Hershey Medical Center, Hershey, USA

**Keywords:** pandemics, specialties, surgical, telemedicine, urology

## Abstract

Introduction*:* During the COVID-19 pandemic, there was a rapid expansion of telemedicine to deliver healthcare. It was speculated that this growth would have a lasting impact on healthcare delivery, but subsequent developments suggest otherwise. This study aims to describe how telemedicine use in urology and other surgical specialties has evolved, four years post-pandemic.

Materials and methods*: *Aretrospective analysis of surgical visits at an academic health system in Hershey, Pennsylvania between July 2020 and July 2024 was conducted. Visits for Urology, General Surgery, OB/GYN, Orthopedics/Rehabilitation, Otolaryngology, and Neurosurgery were categorized as on-site, telephone, or video visits. Basic descriptives, frequencies, and one-way ANOVA were utilized to compare visit types within and between surgical specialties.

Results:A total of 1,514,060 surgical visits occurred in the study period; 4.5% of which were telemedicine. Apart from Urology, all surgical specialties had a large decline in telemedicine use during this interval. Urology had the highest proportion of telemedicine use (11.3%) post-pandemic compared with other surgical specialties, including Neurosurgery (9.9%), General Surgery (8.3%), Otolaryngology (7.2%), OB/GYN (4.1%), and Orthopedics/Rehabilitation (1.2%). Urology used telephone visits (6.7%) more frequently than all other specialties, particularly Otolaryngology (0.3%, p = 0.011) and Orthopedics/Rehabilitation (0.5%, p = 0.049). On average, 88.7% of Urology visits were onsite, while telephone and video comprised 6.7% and 4.6% of visits, respectively.

Conclusions*: *Compared with other surgical specialties, Urology has continued stable use of telemedicine, thereby encouraging continued investments in building infrastructure for this care delivery mechanism. Future efforts should be aimed at developing clinical workflows that integrate telemedicine and advocacy for permanent reimbursement policies that support sustained implementation.

## Introduction

During the COVID-19 pandemic, there was a rapid expansion of telemedicine to deliver healthcare within the United States (U.S.). This growth was primarily driven by the desire to limit COVID-19 exposure and transmission. Notably, in March 2020, the U.S. Centers for Medicare & Medicaid Services allowed for equal reimbursement of in-person and telemedicine visits. Thereafter, other third-party payers quickly followed this precedent, which greatly facilitated the financial incentive and transition to telemedicine within surgical specialties during COVID-19 [1]. It was speculated that this evolution of telemedicine use would have a lasting impact on how healthcare is delivered. Though telemedicine use soared and peaked at the height of the pandemic, the recent closure of multiple commercial virtual care models and recently released financial reports suggest that this may not be the case [2].

The incorporation of telemedicine within surgical specialties has yielded mixed results. Prior to the pandemic, telemedicine in surgery was primarily only used in cases where in-person care was especially difficult due to a lack of access [3]. During the pandemic, surgical practices were found to have been able to adapt quickly, and telemedicine use grew during this time [4,5]. For instance, one randomized trial showed that telemedicine could have a place for post-op appointments, particularly for appointments following minimally invasive surgeries [6]. Another systematic review of telemedicine in surgical care concluded that patients found telemedicine to help save on costs, time, and travel surrounding pre- and post-operative appointments [7]. However, there are a few limitations to telemedicine use specific to surgical practice. Some interactions that are common within surgical specialties, such as consenting, assessing physical findings, breaking bad news, and pre-operative planning, may be less appropriate for the telemedicine modality [8].

Nevertheless, the use and importance of telemedicine within Urology have long been established. The American Urological Association (AUA) has a dedicated AUA Telemedicine Taskforce, consisting of urologists who oversee new telemedicine initiatives within the organization [9]. Prior to the pandemic, telemedicine was already being explored in Urology for patient counseling of urolithiasis, hematuria, and prostate cancer [10-14]. More evidence regarding the efficacy of telemedicine in Urology is still emerging. More recently, post-pandemic, data suggest high patient satisfaction with Urology telemedicine appointments amongst older patients, for prostatectomy follow-up, and for prostate-specific antigen (PSA) surveillance [15]. Another study demonstrated high patient and physician satisfaction with telemedicine appointments for the care of genitourinary malignancies [16].

Understanding how telemedicine is utilized by surgical specialties can provide insight into health care systems regarding optimal future investment into infrastructure creation. This is especially important as healthcare systems emerge from a pandemic that had once made telemedicine necessary. The aim of this study is to describe how telemedicine use in Urology, as well as other surgical specialties, has evolved now four years post-pandemic.

## Materials and methods

Information regarding the surgical visits at a large academic health system between July 2020 and July 2024 was obtained from the Cerner Scheduling Application, Location History, and Revenue Cycle charge tables within the Cerner electronic health record system. Visit types were classified using definitions from the Cerner Scheduling and Revenue Cycle charge datasets. Telephone or video visits were identified as slot appointment types or revenue cycle charges associated with a checked-out Scheduling ID. Video visits were appointments between medical providers and patients through a virtual platform with a camera. Telephone visits were appointments where the medical provider called the patient and spoke to them over the phone. If multiple charges were listed for a visit, a video charge outweighed telephone charge and was counted only once as a video visit. In-person visits were defined as scheduled encounters without an associated telemedicine appointment type or charge. This process was done for all surgical visits, specifically General Surgery, OB/GYN, Orthopedics/Rehabilitation, Otolaryngology, Neurosurgery, and Urology.

Statistical analysis

This data was analyzed retrospectively. Video and telephone visits combined were further categorized as telemedicine visits. Basic descriptives, frequencies, and one-way ANOVA were used to compare the visit types both within and between different surgical specialties. There were no missing data for the primary analytic variables of visit type, date, and specialty, so no imputation was necessary.

## Results

There was a total of 1,514,060 surgical visits across all six surgical subspecialties during the study period. 68,381 (4.5%) of the visits were telemedicine, with 20,151 visits telephone (1.3%) and 48,230 visits video (3.2%) (Table 1).

**Table 1:** In-person, telephone, and video visits across surgical specialties, July 2020 to July 2024.

Specialty	In-Person Visits	In-Person Visits (%)	Telephone Visits	Telephone Visits (%)	Video Visits	Video Visits (%)	Total Visits
General Surgery	154,180	91.70%	4,118	2.45%	9,842	5.85%	168,140
Neurosurgery	66,495	90.14%	1,297	1.76%	5,976	8.10%	73,768
OB/GYN	256,946	95.88%	2,000	0.75%	9,042	3.37%	267,988
Orthopaedics/Rehabilitation	687,330	98.76%	3,605	0.52%	4,997	0.72%	695,932
Otolaryngology	166,252	92.68%	512	0.29%	12,374	6.90%	179,138
Urology	114,476	88.68%	8,619	6.68%	5,999	4.65%	129,094
Total	1,445,679	95.48%	20,151	1.33%	48,230	3.19%	1,514,060

**Figure 1:**
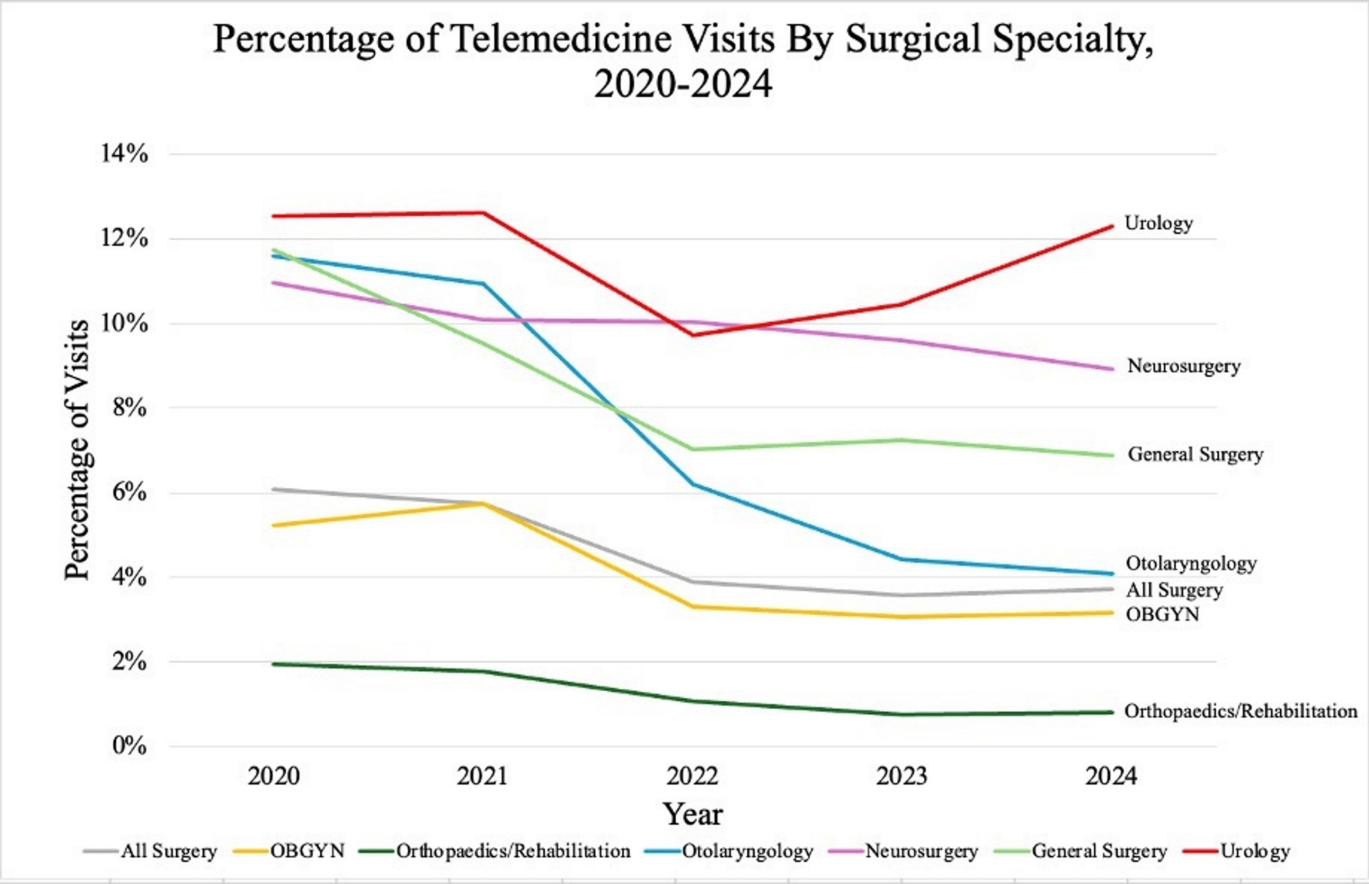
Percentage of telemedicine visits in each surgical specialty July 2020 to July 2024.

With the exception of Urology, all surgical subspecialties experienced a net decline in the use of telemedicine between July 2020 and July 2024. In 2020, the overall percentage of telemedicine use in all surgical specialties combined was 6.1%, which dropped to 3.7% by July 2024. Unlike other surgical subspecialties, however, 12.5% of Urology visits in 2020 were telemedicine, which dropped only slightly to 12.3% in 2024, making Urology the only specialty that did not experience a large decline in telemedicine use overall. Urology had the highest proportion of telemedicine use post-pandemic (11.3%) compared to other surgical specialties, including Neurosurgery (9.9%), General Surgery (8.3%), Otolaryngology (7.2%), OB/GYN (4.1%), and Orthopedics/Rehabilitation (1.2%). Almost all surgical subspecialties peaked in the percentage of telemedicine visits in 2020, with Neurosurgery at 11.0%, Otolaryngology at 11.6%, General Surgery at 11.7%, and Orthopedics/Rehabilitation at 2.0% that year. OB/GYN and Urology peaked in percent telemedicine visits in the year 2021, at 5.7% and 12.6% respectively (Figure 1).

**Figure 2:**
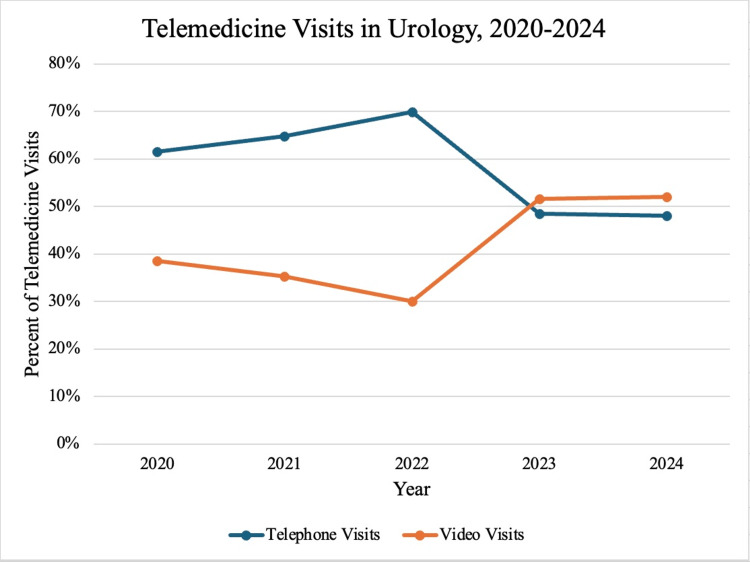
Percent distribution of telephone and video visits within telemedicine visits in Urology, 2020-2024.

A large proportion of Urology’s telemedicine visits were telephone visits. Urology used telephone visits more often than all other specialties (6.7%), in particular, Otolaryngology (0.3%, p = 0.011) and Orthopedics/Rehabilitation (0.5%, p = 0.049). On average, 88.7% of Urology visits occurred onsite during this time period, with telephone and video comprising 6.7% and 4.6% of visits. The majority of Urology telemedicine visits in 2020 were telephone visits (59.0%), but by 2024, the majority of telemedicine visits were video visits (51.9%). (Figure 2). When considering all Urology visits, this is a net decline in telephone visits by 1.8%, and a net increase in video visits by 1.6%.

## Discussion

All surgical specialties, except for Urology, demonstrated an overall decline in the use of telemedicine post-pandemic between July 2020 and July 2024. Some specialties, such as Orthopedics/Rehabilitation, already had low baseline use of telemedicine at the beginning of this study period, starting at 2% and ending at 0.8% by 2024. This aligns with the study by Kuehner et al. of the Kaiser Permanente Northern California surgical services, which revealed that telemedicine use was rare within surgical practices to begin with, even though it rose after COVID restrictions and then declined afterwards [4].

Because surgery is interventional by nature, telemedicine may need further enhancements to improve its applicability [8]. Therefore, though telemedicine may be appropriate for some aspects of surgical care, it cannot fully replace or compensate for the entirety of the scope of practice. For instance, when conducting postoperative visits, full visualization of postoperative incisions, infections, and wounds can be limited, and may even be uncomfortable for patients to share over telemedicine. Yet, the ability to accurately do so is important, especially for patients who may only need reassurance and would have to travel for an inpatient visit. To address this, some medical systems have begun embedding methods for patients to share images for physician review of potentially diagnostic pathology. Additionally, the American College of Surgeons has made statements on how telemedicine can improve within surgical specialties. They advocate for the establishment of best practices for virtual surgical examinations, as well as integration of the enhancements needed to facilitate these practices [5]. Moreover, they suggest that telemedicine within surgery should be seen as an enhancement to in-person care and that joining organizations such as the American Telemedicine Association could provide avenues to advocate for telemedicine within surgical specialties [5]. Given the current low rates of telemedicine use among surgical specialties post-pandemic, it is still unknown how these suggestions may evolve.

Urology was the only specialty with continued stable use of telemedicine between July 2020 and July 2024, in particular, with video visits. Yet, the use of telemedicine within Urology is still limited by many of the issues that pertain to telemedicine in general. Implementation of telemedicine workflows and structures, regardless of specialty, involves time, financial investment, and growing pains while still attempting to deliver quality care [17]. Additionally, though telemedicine can be utilized to improve access to care, it can also worsen existing health disparities. The use of telemedicine requires technological literacy, adequate broadband internet speeds, and ownership of telecommunication, all of which may be less accessible to low-income, rural, and older patients who already face barriers to accessing care [18].

Given that Urology as a surgical specialty has demonstrated past and current investment into telemedicine, looking to alleviate these barriers could facilitate care provision within the field. Some proponents of telemedicine within Urology have called for a conscious, coordinated effort by the government, insurance companies, providers, and hospital systems to make equitable telemedicine services and reimbursement permanent [17]. Additionally, leveraging family help and community organizations to loan or assist with technology for Urology appointments could help make telemedicine more accessible for hard-to-reach populations [18].

This study reports on the surgical visit types of a single academic health system, which may limit the generalizability of these findings. Other practices and healthcare systems may have seen different patterns due to varying existing structures or resources available during the COVID-19 pandemic and afterwards. Furthermore, differing levels of provider and healthcare system comfort with telemedicine prior to the pandemic may further skew these results, both between surgical specialties and between systems. As this was a retrospective analysis of visits, there is the risk of incomplete data due to issues with documentation during the pandemic, as well as selection bias affecting how patients sought care. Finally, as a descriptive analysis, our study was not able to measure patient and provider outcomes, and there may be confounders that influenced the trends observed. In particular, this study did not account for patient outcomes or satisfaction, which are key variables in the success of telemedicine. Future studies can investigate these outcomes to provide further details on what factors influence how telemedicine is utilized within surgical specialties. 

## Conclusions

Telemedicine use in surgical specialties has either remained low or diminished four years post-pandemic. It remains to be seen what role telemedicine will play within surgical specialties in the future. However, compared with other surgical specialties, Urology has continued stable use of telemedicine both pre- and post-pandemic, and the existing literature appears to support the use of telemedicine within Urology. Therefore, it is worthwhile to continue investing in and building infrastructure for this care delivery mechanism to enhance urologic care.
